# Epidemiological feature of imported malaria in Taiwan during the 2014-to-2020 period

**DOI:** 10.1097/MD.0000000000041321

**Published:** 2025-01-17

**Authors:** Fu-Huang Lin, Yu-Ching Chou, Chi-Jeng Hsieh, Yao-Ching Huang, Chia-Peng Yu

**Affiliations:** a School of Public Health, National Defense Medical Center, Taipei City, Taiwan; b Department of Healthcare Administration, Asia Eastern University of Science and Technology, New Taipei City, Taiwan; c Department of Chemical Engineering and Biotechnology, National Taipei University of Technology (Taipei Tech), Taipei, Taiwan; d Department of Medical Research, Tri-Service General Hospital, National Defense Medical Center, Taipei City, Taiwan.

**Keywords:** epidemiology, immigrant, imported case, malaria, traveler

## Abstract

Although the World Health Organization (WHO) certified Taiwan as being malaria-free in 1965, there are reports of a few imported cases each year by travelers who visit malaria-endemic areas. This study examined the epidemiology of imported malaria cases in Taiwan from 2014 to 2020, utilizing national surveillance data from the Taiwan Centers for Disease Control. Malaria cases were confirmed through the application of standard laboratory methods. Passenger data came from the Tourism Bureau, Ministry of Transportation and Communication, Taiwan (TBMTC). All data were analyzed using SPSS version 21. The analysis included a dataset comprising 64 cases of imported malaria. Of the total cases, 77.8% were acquired from Africa, and 17.5% from Asia. *Plasmodium falciparum* was responsible for more than half (57.1%) of the cases, *Plasmodium vivax* malaria for 25.4% of cases, *Plasmodium malariae* malaria for 6.3%, *Plasmodium ovale* malaria for 4.8%, and unspecified pathogen malaria for 6.3% of the cases. Majority of the patients were male (75%) and were predominantly aged 20 to 59 years (70.3%). Most cases of imported malaria occurred during the fall season, and 51.6% of cases occurred in 8 cities during the period of 2014 to 2020. No evidence exists to indicate that indigenous malaria transmission occurs in Taiwan. *Anopheles minimus* was found in 4 cities (counties), namely Tainan City and Pingtung County in Southern Taiwan; Hualien County and Taitung County in Eastern Taiwan. The findings of this study highlight the necessity for robust surveillance systems, effective vector control measures, and targeted interventions for travelers and immigrants to prevent malaria outbreaks and maintain Taiwan’s malaria-free status.

## 1. Introduction

Malaria is caused by 5 single-cell eukaryotic malarial parasites of the *Plasmodium* spp., namely *P falciparum*, *P ovale*, *P malariae*, *P vivax*, and *P knowlesi*, which transmitted via the bite of female *Anopheles* mosquitoes.^[1]^ Malaria remains a significant global health concern, attracting substantial scientific interest. Despite a 25.9% reduction in malaria cases from 2006 to 2016,^[2,3]^ an estimated 216 million individuals contracted the disease, and 719,600 died from it in 2016.^[4]^ This resulted in malaria ranking as the sixth leading cause of mortality on a global scale in that year.^[3]^

Malaria in non-endemic countries can also cause death among local residents due to yearly imported cases.^[5]^ The changing risk of malaria infection has been influenced by shifts in travel behaviors, migration patterns, and the development of drug resistance.^[6,7]^ To reduce the likelihood of infection, travelers should implement preventive measures, including wearing long-sleeved clothing, using insect repellents, sleeping under insecticide-treated nets, and taking appropriate antimalarial medication.^[8–10]^ The efficacy of these measures is contingent upon travelers’ awareness and understanding of the risks associated with malaria.^[11]^

Despite global efforts to elimination malaria, reemergence may still occur in areas where it has been previously eliminated. This public health concern reminds us that as long as there are malaria-transmitting mosquito vectors and suitable climatic conditions, the spread of the disease can become rapid. Therefore, a good epidemiological surveillance system allows you to quickly assess the presence of outbreaks to carry out control actions.^[12]^ It is therefore probable that malaria will be eliminated and sustained even in areas with a low infection rate or reduced health quality due to human development, or in areas that are geographically isolated, cross-border mobility restricted, and have limited parasite import. This is likely to be the case once malaria elimination is achieved.^[13–17]^

Taiwan’s subtropical climate, situated at 23° 4’N, 121° 0’E, is distinguished by a considerable range of temperatures and a pervasive prevalence of high humidity.^[18]^ Imported cases continue to occur every year, causing serious health threats to the people of Taiwan.^[18]^ In order to ascertain the risk factors and epidemiological characteristics of malaria, we conducted an analysis of all reported malaria cases in Taiwan between 2014 and 2020. The distribution of imported cases included in the study was assessed and compared to determine whether these data analysis will help improve the existing monitoring system and formulate suggestions for health policies.

## 2. Materials and Methods

### 2.1. Ethical policy

The ethical policy of this study was similar to 1 used by Holland et. al. 2021.^[19]^ This study gathered data from the electronic health records of the Taiwan Centers for Disease and Control (TCDC).^[20]^ Thus, institutional review board approval and informed consent were not required due to the acquisition of data from secondary sources in this study. All data were de-identified, and the study included information and analysis of open dataset sources that were freely available in the public domain, where the data were properly anonymized.^[21–23]^

## 3. Surveillance of malaria in Taiwan

In 1990, the TCDC established the National Notifiable Disease Surveillance System (TNNDSS)^[24]^ for streamlined reporting of malaria cases. In Taiwan, malaria is a nationally notifiable disease that legally mandates doctors to report any confirmed case within 24 hours using a software developed by TCDC that is electronically forwarded to the TCDC.^[25]^ According to a survey conducted in Taiwan,^[26]^ more than 84% of doctors reported malaria cases to the TCDC upon confirmation of the disease. Upon receipt of the report, the TCDC assigns an epidemiological team consisting of a field epidemiologist, an entomologist, and a public health nurse to follow up on the patients, verify the diagnosis, and obtain complete patient information. Follow-ups included face-to-face interviews, telephone calls, communications with healthcare providers, and interviews with patients. The information collected included patient age, gender, area of residence, geographical location of exposure, contact details, and travel history.^[27]^

## 4. Travel data

Passenger data came from the Tourism Bureau, Ministry of Transportation and Communication, Taiwan (TBMTC).^[28]^ Data from the TBMTC included the number of tourists entering Taiwan from malaria-endemic countries from 2014 to 2020. The number of tourists from various countries visiting Taiwan is based on immigration/ disembarkation cards and travel agency reports completed for immigration and tourism purposes.

## 5. Mosquito data

The survey data on malaria vectors in Taiwan were obtained from TCDC.^[29]^ The monthly survey was conducted in 2 to 3 villages each month from January to December every year between 2014 and 2020. During each visit, dippers with a diameter of 14 cm, were used to conduct larval surveys along streams and ditches around or inside the surveyed villages. All collected mosquitoes were stored in dry ice boxes and brought back to the laboratory for species identification. The mosquitoes were maintained at −20 °C for species identification.^[29,30]^

## 6. Definitions

Malaria cases were defined as patients confirmed to be infected with malarial parasites in the laboratory between 2014 and 2020. Laboratory confirmation was established when the malaria parasite was detected on microscopic examination of the patient’s blood smear or subsequently confirmed and identified by polymerase chain reaction.^[31,32]^ A region is deemed to have successfully eradicated malaria when there is no local transmission of the disease and the number of indigenous cases has reached zero, irrespective of the occurrence of occasional imported cases.^[33]^

## 7. Statistical analysis

This study included a retrospective historical study of malaria cases imported to Taiwan from 2014 to 2020. We identified malaria cases by year from 2014 to 2020 from the database and examined the differences and trends in the distribution of their epidemiological characteristics. Descriptive data are shown as numbers where appropriate. All statistical analyses were performed using SPSS software (IBM SPSS Statistics 21; Asia Analytics Taiwan Ltd., Taipei, Taiwan). All statistical tests were 2-sided with an α level of 0.05. P-values < 0.05 were considered statistically significant.

## 8. Results

**Figure 1.**
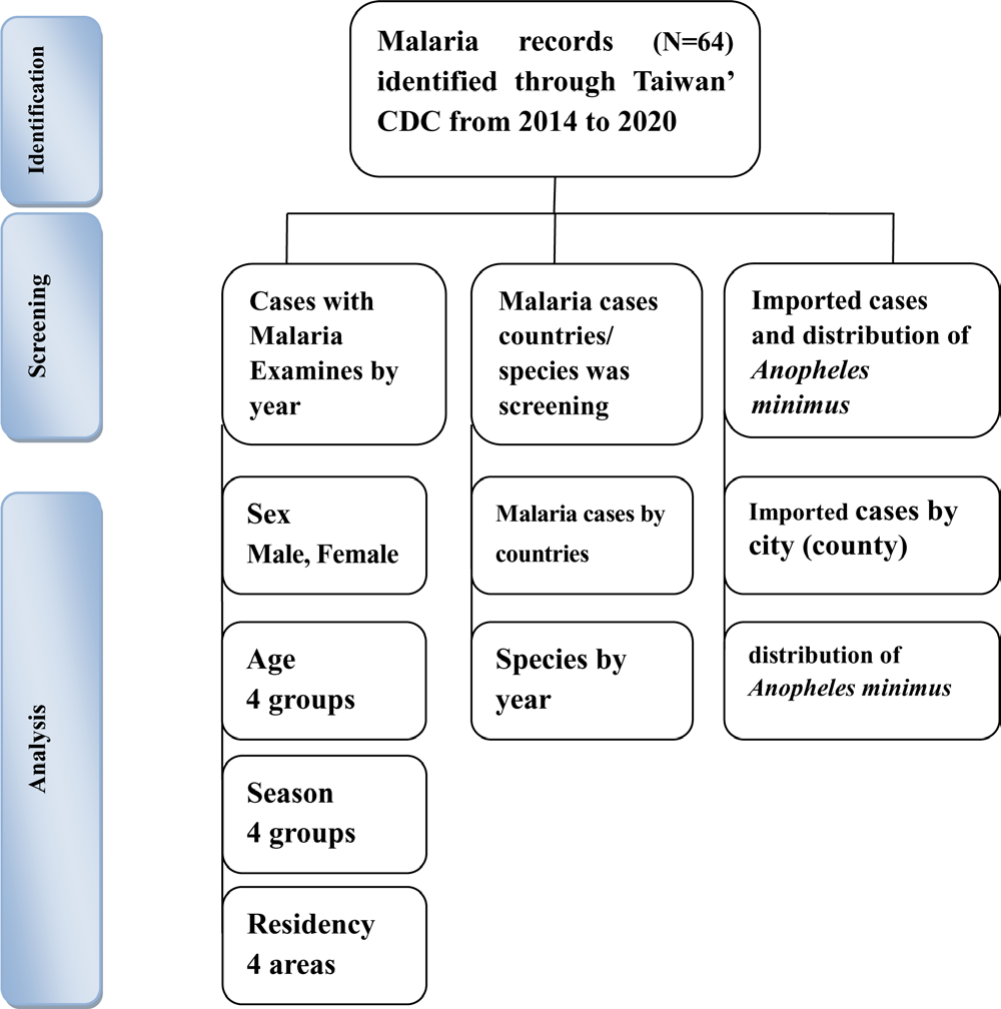
Flowchart of the study sample selection from Taiwan Centers for Disease Control Database in Taiwan (2014–2020).

The study sample selected from the database of the TCDC from 2014 to 2020 is shown in Figure 1. A total of 64 malaria cases were reported in Taiwan during this period. Table 1 shows the epidemiological characteristics of the patients. Of the 64 patients, 48 were men (75.0%), and the male-to-female ratio was 3:1. There were 35 patients aged 20 to 39 (54.7%), 28 infected during autumn (43.8%), and 33 came from northern China (51.6%). The number of malaria cases reached its highest point in 2014, with 19 cases reported, while the lowest number of cases, 2, was recorded in 2020. All cases documented during the study period were imported, with no evidence of local transmission or secondary infections. The ratio of locally acquired to imported cases was 0:64. This data unequivocally meets the minimum criteria for certification of “malaria elimination.”^[34]^

**Table 1:** Epidemiological feature of imported cases with malaria from 2014 to 2020, Taiwan.

Variable	Year*							
	Overall N = 64	2014 N = 19	2015 N = 9	2016 N = 13	2017 N = 7	2018 N = 7	2019 N = 7	2020 N = 2
Importation rate per 1,000,000	1.1	1.9	0.9	1.2	0.7	0.6	0.6	1.5
Sex								
Male	48	15	8	9	6	5	4	1
Female	16	4	1	4	1	2	3	1
Age group								
<20	7	3	0	3	0	1	0	0
20 to 39	35	8	8	8	4	4	2	1
40 to 59	15	5	0	1	3	1	5	0
≥60	7	3	1	1	0	1	0	1
Season								
Spring	9	4	2	2	1	0	0	0
Summer	17	4	3	2	4	2	2	0
Fall	28	8	3	6	2	4	4	1
Winter	10	3	1	3	0	1	1	1
Residency								
Northern	33	11	2	9	2	4	4	1
Central	11	2	4	1	1	1	1	1
Southern	15	5	2	1	3	2	2	0
Eastern	5	1	1	2	1	0	0	0

* No cases of deaths due to malaria were reported during 2014 to 2020.

Using the statistical data from TBMTC, the number of tourists coming from malaria-epidemic countries can be estimated more accurately. During the study period, the annual malaria import rate changed significantly, from 19 in 2014 to 7 in 2019, resulting in a 68% decrease. The annual average overseas malaria import rate is 1.1 per 1 million people (range 0.6–1.9) (Table 1). This finding also meets the minimum requirement to maintain the interruption of malaria transmission, which is a < 0.2 infection import rate per 1000 people.^[35]^ Majority (76.6%) of the cases were imported from Africa, followed by Asia (17.2%), and Oceania (4.7%). Of the 64 patients, 6 were overseas tourists from Nigeria (92/129), 5 were from Uganda, 5 were from India, and the remaining were from different countries (with <5 cases per country) (Table 2). The number of imported malaria cases ranged from 2 to 19 per year. Among the 64 reported cases, 56.3% were due to *P falciparum* and 25% were due to *P vivax*. Moreover, there were no confirmed cases of *P knowlesi* (Table 3). There was no reported mortality in the known cases.

**Table 2:** The cases with malaria reported by region/countries of likely acquisition, 2014 to 2020 in Taiwan.

Countries	Year							
	Overall N = 63	2014 N = 19	2015* N = 8	2016 N = 13	2017 N = 7	2018 N = 7	2019 N = 7	2020 N = 2
Asia (N = 11)								
India	5	2	2			1		
Vietnam	1	1						
Indonesia	2			1	1			
Thailand	2			2				
Malaysia	1							1
Oceania (N = 3)								
Solomon Islands	2					1	1	
Papua New Guinea	1						1	
Africa (N = 49)								
Côte d’Ivoire	4	3			1			
*Burkina Faso*	4	3			1			
*Nigeria*	6	2	1		2		1	
Ghana	3	2		1				
Central African Republic	2	1	1					
Cameroon	2	1	1					
Ethiopia	4	1				1	2	
Kenya	2	1				1		
Gabon	1	1						
Congo	3		2				1	
Malawi	3	1	1	1				
Equatorial Guinea	1				1			
South Africa	1				1			
Uganda	5					3	1	1
*Gambia*	4			4				
*Sierra Leone*	2			2				
Angola	1			1				
Mozambique	1			1				

* One data was lost.

**Table 3:** The species of malaria reported by years of likely acquisition, 2014 to 2020 in Taiwan.

Pathogens*	Year							
	Overall N = 63	2014 N = 19	2015† N = 8	2016 N = 13	2017 N = 7	2018 N = 7	2019 N = 7	2020 N = 2
*P falciparum*	36	10	5	10	5	3	3	
*P vivax*	16	4	2	3	1	2	3	1
*P malariae*	4	2				1	1	
*P ovale*	3	1	1					1
Unspecified	4	2			1	1		

* No cases with *P knowlesi* infection.

† One data was lost.

From 2014 to 2020, only 14 (63.6%) of 22 counties of Taiwan and cities reported imported malaria cases from abroad, and no secondary transmission was reported. There were 49 cases (76.6%) in 6 counties and cities, but *A minimus* was found in only 4 of them, namely Tainan City and Pingtung County in Southern Taiwan and Hualien County and Taitung County in the Eastern region. In general, imported cases tend to gather in the absence of counties and cities containing *A minimus* (Table 4).

**Table 4:** Correlation between imported malaria cases and *Anopheles* mosquitoes in Taiwan (2014–2020).

County (City)	Imported cases (N = 64)	Areas where malaria mosquitoes appeared*
Northern		
New Taipei city	12	–
Taipei city	11	–
Keelung city	1	–
Yilan county	1	–
Taoyuan city	6	–
Hsinchu city	2	–
Central		
Taichung city	6	–
Changhua county	2	–
Nantou county	3	–
Southern		
Yunlin county	1	–
Tainan city	7	X
Kaohsiung city	7	–
Pingtung county	0	X
Eastern		
Hualien county	4	X
Taitung county	1	X

* X symbol indicated that *Anopheles minimus* mosquitoes appeared.

## 9. Discussion

This study employed an analysis of long-term surveillance data to investigate the epidemiological characteristics of 64 imported malaria cases in Taiwan between 2014 and 2020. Four significant epidemiological features were identified. First, the imported malaria cases were predominantly concentrated in the northern regions, accounting for 33 cases (51.6%) of the total. Of these cases, 11 were identified in Taipei City and 12 in New Taipei City. Second, the imported malaria cases mostly affected male travelers and those aged 20 to 59 years. Third, a seasonal pattern was discernible in imported malaria cases, with the peak incidence occurring during the summer and autumn months, accounting for 70.3% of all cases. Fourth, among the imported malaria cases, *P falciparum* was the dominant pathogen, and there were no reported deaths. In Taiwan, imported malaria cases originated from African and Asian countries, and majority of the cases (77.8%) came from malaria-endemic countries in Africa. The imported cases were related to travel to epidemic areas (such as Nigeria), mainly for business or visiting friends and relatives. The mode of imported malaria was related to the transmission intensity of patients’ visits to sites, the number of tourists in each epidemic area, the population and ethnic background of tourists, activities carried out during travel, the type of accommodation they use, and their compliance with prevention.^[36]^ In general, our findings support the results reported in previous research and highlight the role of tourists coming from Africa in importing malaria transmission in countries where malaria is not epidemic (such as Taiwan).^[37]^ In light of the fact that all malaria cases documented during the study were imported and no secondary cases were identified, the ratio of local to imported cases was determined to be 0:64. This data unequivocally meets the minimum criteria for certification of malaria elimination.^[34]^

Malaria was endemic in Taiwan for much of the 19th and 20th centuries, reaching epidemic proportions in 1952 with an estimated 1.2 million cases.^[15,38]^ In the late 1960s, a multifaceted approach was implemented to combat malaria, which included improvements in housing and socioeconomic conditions, environmental sanitation, and the intensive use of dichloro-diphenyl-trichloroethane.^[39,40]^ This comprehensive strategy resulted in a significant reduction in malaria transmission, which led to Taiwan being certified as a malaria-free area by the World Health Organization (WHO) in November 1965.^[41]^ Since then, the TCDC has continuously maintained the malaria case monitoring system for early detection and warning of overseas immigration cases, and observed the drug resistance pattern of antimalarial drugs, acknowledging that malaria transmission may be reintroduced. Over the past 40 years, Taiwan has been reporting confirmed malaria cases imported from overseas. Moreover, in Taiwan, all malaria cases were confirmed to be imported.^[42]^ In the past 2 decades, the importation of malaria has been a growing health concern in Taiwan and Western countries. The increase in imported malaria cases may be attributed to a number of factors, including an increase in tourism to tropical regions and immigration from malaria-endemic countries.^[11,43,44]^ Despite the absence of indigenous malaria cases in Taiwan for several decades, the risk of reemergence persists due to the country’s climate, growing populations in mosquito-prone areas, and increased travel and immigration from malaria-endemic regions. Greece, which has been free of malaria since 1974, has experienced isolated cases of indigenous malaria on a few occasions.^[45]^ Therefore, residents in Taiwan should be aware of the vulnerability of an outbreak malaria resurgence. This study concludes that Taiwan’s current medical care, mosquito control, and public health infrastructure remain intact; however, the slight possibility of another malaria outbreak in Taiwan warrants continuous monitoring efforts.

Another possible explanation for the decline in malaria cases in Taiwan includes the efforts of clinicians and public health experts around the world in reducing the spread of malaria in endemic countries. The implementation of malaria prevention and treatment strategies has expanded the reach of effective interventions globally, resulting in a decline in malaria cases.^[37]^ It is possible that the number of malaria cases in Taiwan may continue to decline as a result of sustained preventive and treatment efforts to curb endemic malaria transmission.

Between 1987 and 2006, the UK saw 191 (0.5%) fatalities among 39,302 confirmed malaria cases. The age and infection in non-endemic regions were linked to a higher risk of fatal outcomes.^[46]^ In 2017, the TCDC reported around 7 imported malaria cases, which is a 63% drop from 2014 (19 cases). There were no deaths during the study period. There are a few reasons for this drop and no deaths, including changes in how people travel around the world, less malaria, new ways of tracking cases, and better ways to prevent and treat it. It is unlikely that the decrease is due to less travel between Taiwan and malaria-endemic countries, as visitor numbers increased by about 12% from 2014 to 2019.^[28]^

The highest risk of malaria infection among travelers has been documented in Saharan Africa and Papua New Guinea, followed by the middle of the Indian subcontinent, with the lowest risk reported in Southeast Asia and Latin America.^[47–49]^ However, the number of exported malaria cases from these regions varied widely.^[50–52]^ Most malaria reports are based on nationally reported data; however, most reports fail to account for the total number of travelers, leading to difficulty in assessing the risk of overseas migration based solely on these case numbers. For example, malaria rates among travelers vary quite a bit from country to country. Ghana has as many as 714 cases per 100,000 travelers, while Thailand rate is much lower at 2.5 per 100,000.^[53]^ This study shows that Africa (76.6%), followed by Asia, represents the largest number of countries of origin for imported malaria cases outside Taiwan. These findings clearly confirm that the risk of malaria infection among travelers comes from entering and leaving malaria-endemic countries or regions. The data presented here enables us to conduct a risk analysis of individuals traveling to malaria-endemic areas.

*Plasmodium falciparum* is the most frequently reported pathogenic parasite of human malaria.^[54,55]^ Similarly, *P falciparum* was the most predominant pathogen among the imported malaria cases in this study, which conforms with those reported in other studies.^[56,57]^ In light of Taiwan’s successful elimination of malaria, clinicians and nursing staff in the country may lack sufficient knowledge needed to prevent and treat falciparum malaria. This study suggests that the education and training of medical staff must be strengthened in this regard. Moreover, at this stage, the import of malaria is a major public health challenge in Taiwan. The implementation of a thorough epidemic prevention plan can ensure timely diagnosis, confirmation of cases, and correct classification and treatment, so as to ensure that imported malaria cases do not spread locally. Therefore, it is crucial that public health officials, healthcare providers, immigration and quarantine services, and law enforcement work together to keep imported malaria under control.^[55]^

It is reported that serious malaria cases result from the failure of health personnel to identify the disease at an early stage.^[58]^ Furthermore, the early, accurate, and timely detection and clinical treatment of malaria play a key role in reducing the incidence and mortality of malaria, delaying drug resistance, and improving malaria control. It is necessary to ensure the implementation of universal diagnostic and testing procedures for all suspected malaria cases.^[59]^ It is therefore imperative that the early detection, diagnosis, and management of imported malaria be given priority.^[60]^ Moreover, healthcare professionals must be aware that imported cases, particularly those originating from non-African regions, require prompt diagnosis and treatment. However, medical staff in Taiwan may have insufficient skills needed to detect imported malaria from abroad; thus, better clinical training is needed for the current situation.^[61]^

Due to the presence of *Anopheles* mosquitoes and favorable environmental conditions, Taiwan still faces the risk of malaria re-emergence. *A minimus* serves as the primary vector for malaria transmission in Taiwan.^[30]^ It is important for clinicians to be aware that, in addition to *P falciparum*, other *Plasmodium* species can also cause significant health complications. Therefore, when providing pretravel advice, healthcare providers should emphasize the importance of preventing all types of malaria infection.^[47]^ When entering Taiwan, passengers with fever or respiratory symptoms should take the initiative to fill in the “entry health declaration,” and undergo thorough TCDC border quarantine measures, timely diagnosis, and differential diagnosis of fever patients with a travel history in malaria-endemic areas. Malaria often presents with a nonspecific constellation of symptoms, most commonly fever. Additional symptoms may include headache, chills, excessive sweating, backache, muscle pain, diarrhea, nausea, vomiting, and cough. Despite optimal clinical management, delays in diagnosis and treatment can lead to serious complications. While the number of imported malaria cases in Taiwan has decreased in recent years,^[62]^ the risks to both tourists and residents remain evident. Therefore, this study suggests that individuals planning to travel to malaria-endemic countries or regions should receive health-related travel advice, and clinicians may take the initiative and pay attention to and evaluate the epidemiological characteristics of returning tourists, such as fever and overseas travel history, to improve the diagnosis rate of the disease.

New data from the WHO reveal that the COVID-19 pandemic has disrupted malaria services, leading to a marked increase in cases and deaths. According to WHO’s latest World malaria report, there were an estimated 241 million malaria cases and 627,000 malaria deaths worldwide in 2020.^[63]^ This study also found that the overseas import rate of malaria declined in Taiwan (excluding the 2020 border blockade) from 2014 to 2019. However, the overseas import rate of malaria increased in Taiwan 2020. This study was similar with previous study.^[63]^ This study infers that the reason may be the covid-19 epidemic. Although the number of tourists has decreased, the spread of vector-borne infectious diseases (such as malaria) would still peak in 2020, resulting in an increase in the overseas imported rate.

The advantages of our study are the input of case data and the distribution of malaria mosquitoes outside Taiwan. The TCDC relies on the reports of each county and city to compile the national malaria case data. As a result, health care providers promptly report all malaria cases to enable assessment and tracking of annual trends. However, our study has several limitations, including the inherent uncertainty of malaria data and traveler statistics. Given the prevalence of multi-country travel within the region, malaria surveillance reports may not accurately reflect the true country of infection. In addition, cases that manifest symptoms or receive treatment abroad may not be captured in Taiwan’s national surveillance system, further compromising data accuracy. Underreporting of malaria cases remains a global challenge,^[44]^ which may lead to an underestimation of the true incidence of imported malaria in Taiwan. This study indicated that, due to the necessity of copyright authorization from commercial companies for the use of images, the regions of the country were not mapped and highlighted according to the presence of the vector.

## 10. Conclusions

To sum up, from 2014 to 2020, the overseas import rate of malaria declined in Taiwan (excluding the 2020 border blockade). All confirmed imported malaria cases were associated with overseas imports. However, the diseases causing outbreak transmission are extremely limited, and no outbreak malaria transmission has been found in Taiwan. Effective elimination of malaria in Taiwan requires timely identification and diagnosis of all malaria cases, providing good environmental management, vector control, and health education for tourists and immigrants coming from malaria-endemic areas. The combination of these measures will ensure that Taiwan maintains its goal of eradicating malaria-affected areas.

## Acknowledgments

The authors are grateful to all our colleagues in the School of Public Health in National Defense Medical Center (Taiwan) for their help in the collection of government data and technical assistance.

## Author contributions

**Conceptualization:** Yu-Ching Chou, Yao-Ching Huang, Chia-Peng Yu.

**Data curation:** Fu-Huang Lin, Yu-Ching Chou.

**Formal analysis:** Fu-Huang Lin, Chi-Jeng Hsieh, Chia-Peng Yu.

**Methodology:** Yu-Ching Chou, Chi-Jeng Hsieh, Yao-Ching Huang.

**Writing – original draft:** Fu-Huang Lin, Chia-Peng Yu.

**Writing – review & editing:** Fu-Huang Lin, Chia-Peng Yu.

## References

[1] Phillips MA Burrows JN Manyando C van Huijsduijnen RH Van Voorhis WC Wells TNC. Malaria. Nat Rev Dis Primers. 2017;3:17050. 28770814 10.1038/nrdp.2017.50

[2] Iqbal J Al-Awadhi M Ahmad S. Decreasing trend of imported malaria cases but increasing influx of mixed *P. falciparum* and *P. vivax* infections in malaria-free Kuwait. PLoS One. 2020;15:e0243617. 33306727 10.1371/journal.pone.0243617 PMC7732060

[3] GBD 2016 Causes of Death Collaborators. Global, regional, and national age-sex specific mortality for 264 causes of death, 1980-2016: a systematic analysis for the Global Burden of Disease Study 2016. Lancet. 2017;390:1151–210. 28919116 10.1016/S0140-6736(17)32152-9 PMC5605883

[4] World Health Organization. World malaria report 2016 [M]. Geneva: WHO Press; 2017: 17–18.

[5] World Malaria Report 2018. World Health Organization; Geneva, Switzerland: 2018. https://iris.who.int/bitstream/handle/10665/275867/9789241565653-eng.pdf. Accessed March 1, 2022.

[6] Smith AD Bradley DJ Smith V. Imported malaria and high risk groups: Observational study using UK surveillance data 1987–2006. BMJ. 2008;337:a120. 18599471 10.1136/bmj.a120 PMC2453297

[7] MacPherson DW Gushulak BD Baine WB. Population mobility, globalization, and antimicrobial drug resistance. Emerg Infect Dis. 2009;15:1727–32. 19891858 10.3201/eid1511.090419 PMC2857230

[8] Lalani T Yun H Tribble D. A comparison of compliance rates with anti-vectorial protective measures during travel to regions with dengue or chikungunya activity, and regions endemic for *Plasmodium falciparum* malaria. J Travel Med. 2016;23:taw043. 27378367 10.1093/jtm/taw043 PMC4939934

[9] Kuna A Gajewski M Stańczak J. Evaluation of knowledge and use of the malaria prevention measures among the patients of the Department of tropical and parasitic diseases university center of maritime and tropical medicine, Gdynia, based on a questionnaire performed in the years 2012-20. Przegl Epidemiol. 2017;71:33–44. 28654740

[10] Maia MF Kliner M Richardson M Lengeler C Moore SJ. Mosquito repellents for malaria prevention. Cochrane Database Syst Rev. 2018;2:CD011595. 29405263 10.1002/14651858.CD011595.pub2 PMC5815492

[11] Toovey S Jamieson A. Rolling back malaria: how well is Europe doing? Travel Med Infect Dis. 2003;1:167–75. 17291910 10.1016/j.tmaid.2003.09.004

[12] Mendis K Rietveld A Warsame M Bosman A Geenwood B Wernsdorfer WH. From malaria control to eradication: the WHO perspective. Trop Med Int Health. 2009;14:802–9. 19497083 10.1111/j.1365-3156.2009.02287.x

[13] Eliminating Malaria. World Health Organization, Geneva, Switzerland: 2016. https://iris.who.int/bitstream/handle/10665/205565/WHO_HTM_GMP_2016.3_eng.pdf. Accessed March 1, 2022.

[14] Cohen JM Moonen B Snow RW Smith DL. How absolute is zero? An evaluation of historical and current definitions of malaria elimination. Malar J. 2010;9:213. 20649972 10.1186/1475-2875-9-213 PMC2983111

[15] From malaria control to malaria elimination: a manual for elimination scenario planning. World Health Organization; Geneva, Switzerland: 2014. https://iris.who.int/bitstream/handle/10665/112485/9789241507028_eng.pdf?sequence=1. Accessed March 1, 2022.

[16] Feachem RG Phillips AA Hwang J. Shrinking the malaria map: progress and prospects. Lancet. 2010;376:1566–78. 21035842 10.1016/S0140-6736(10)61270-6 PMC3044848

[17] Moonen B Cohen JM Snow RW. Operational strategies to achieve and maintain malaria elimination. Lancet. 2010;376:1592–603. 21035841 10.1016/S0140-6736(10)61269-X PMC3037542

[18] Lin CY Chang K Chang CJ. Questionnaire-based analysis of adverse events and compliance with malaria chemoprophylaxis in Taiwan. J Pers Med. 2023;13:179. 36836413 10.3390/jpm13020179 PMC9967687

[19] Holland KM Jones C Vivolo-Kantor AM. Trends in US emergency department visits for mental health, overdose, and violence outcomes before and during the COVID-19 Pandemic. JAMA Psychiatry. 2021;78:372–9. 33533876 10.1001/jamapsychiatry.2020.4402 PMC7859873

[20] National Infectious Disease Statistics System in Taiwan. 2022. https://nidss.cdc.gov.tw/. Accessed June 1, 2022.

[21] Lin FH Chen BC Chou YC. The epidemiology of entamoeba histolytica infection and its associated risk factors among domestic and imported patients in Taiwan during the 2011-2020 Period. Medicina (Kaunas). 2022;58:820. 35744083 10.3390/medicina58060820 PMC9228342

[22] Lin FH Chou YC Chien WC Chung CH Hsieh CJ Yu CP. Epidemiology and risk factors for notifiable scrub typhus in Taiwan during the period 2010-2019. Healthcare (Basel). 2021;9:1619. 34946346 10.3390/healthcare9121619 PMC8701143

[23] Lin FH Chen BC Chou YC Hsieh CJ Yu CP. Incidence and risk factors for notifiable typhoid and paratyphoid in Taiwan during the period 2011-2020. Healthcare (Basel). 2021;9:1316. 34682996 10.3390/healthcare9101316 PMC8544365

[24] Taiwan Centers Disease and Control. 2024. https://nidss.cdc.gov.tw/nndss/disease?id=0701. Accessed September 1, 2024.

[25] Notifiable Infectious Disease Statistical System. Taiwan Centers for Disease Control; Taipei, 2022. https://nidss.cdc.gov.tw. Accessed March 1, 2022.

[26] Tan HF Yeh CY Chang HW Chang CK Tseng HF. Private doctors’ practices, knowledge, and attitude to reporting of communicable diseases: a national survey in Taiwan. BMC Infect Dis. 2009;9:11. 19178741 10.1186/1471-2334-9-11 PMC2642829

[27] Guideline of National Notifiable Disease. Taiwan Centers for Disease Control; Taipei, 2022. https://www.cdc.gov.tw. Accessed March 14, 2022.

[28] Outbound Departures of Nationals of Taiwan by Destination, 2014–2020. Tourism Bureau, Ministry of Transportation and Communication, Taiwan, 2022. https://stat.taiwan.net.tw/statistics/year/inbound/gender. Accessed March 1, 2022.

[29] Malaria in Taiwan. Centers for Disease Control, Taiwan, 2022. https://www.cdc.gov.tw. Accessed March 1, 2022.

[30] Chang MC Teng HJ Chen CF Chen YC Jeng CR. The resting sites and blood-meal sources of *Anopheles minimus* in Taiwan. Malar J. 2008;7:105. 18538036 10.1186/1475-2875-7-105 PMC2435115

[31] Manual of Standard Operation Procedure of Communicable Diseases. Taiwan Centers for Disease Control; Taipei, 2011. https://www.cdc.gov.tw/En/File/Get/4_0CtIOox8gJBRNzs2QI2A. Accessed March 1, 2022.

[32] McClure EM Meshnick SR Mungai P. The association of parasitic infections in pregnancy and maternal and fetal anemia: a cohort study in coastal Kenya. PLoS NeglTrop Dis. 2014;8:e2724. 10.1371/journal.pntd.0002724 PMC3937317 24587473

[33] Greenwood B Zongo I Dicko A Chandramohan D Snow RW Ockenhouse C. Resurgent and delayed malaria. Malar J. 2022;21:77. 35264158 10.1186/s12936-022-04098-6 PMC8905818

[34] Smith DL Hay SI. Endemicity response timelines for *Plasmodium falciparum* elimination. Malar J. 2009;8:87. 19405974 10.1186/1475-2875-8-87 PMC2686731

[35] Crowell V Hardy D Briët O Chitnis N Maire N Smith T. Can we depend on case management to prevent re-establishment of P. falciparum malaria, after local interruption of transmission? Epidemics. 2012;4:1–8. 22325009 10.1016/j.epidem.2011.10.003

[36] Freedman DO Chen LH Kozarsky PE. Medical considerations before international travel. N Engl J Med. 2016;375:247–60. 27468061 10.1056/NEJMra1508815

[37] Stäger K Legros F Krause G. Imported malaria in children in industrialized countries, 1992–2002. Emerg Infect Dis. 2009;15:185–91. 19193261 10.3201/eid1502.080712 PMC2657617

[38] Malaria Eradication in Taiwan. Department of Health, Taiwan: 2005. https://www.cdc.gov.tw/InfectionReport/Info/SVtdjRgESOT_EwbAhjIJ4g?infoId=mrl8S_96ADvSpl0j2kwX9A. Accessed March 1, 2022.

[39] Malaria control and eradication in Taiwan. https://apps.who.int/iris/bitstream/handle/10665/265315/PMC2537726.pdf?sequence=1&isAllowed=y. Accessed March 1, 2022.

[40] Chen HH Chen AL. Indoor residual spraying of DDT for malaria control. Amer J Public Health. 2009;99:1350–1. 10.2105/AJPH.2009.163717 PMC2707477 19542027

[41] Liang KC. Historical review of malaria control program in Taiwan. Gaoxiong Yi Xue Ke Xue Za Zhi. 1991;7:271–7. 2056561

[42] Tang JS Chen CL Ko WC Chuang CC. Imported malaria in southern Taiwan from 1991 to 2002: a single hospital’s experience. Kaohsiung J Med Sci. 2003;19:398–405. 12962427 10.1016/S1607-551X(09)70483-8 PMC11917622

[43] UNWTO World Tourism Barometer and Statistical Annex, 2019. United Nation World Tourism Organization. https://www.e-unwto.org/toc/wtobarometereng/17/4. Accessed March 1, 2022.

[44] Jelinek T Schulte C Behrens R. Imported falciparum malaria in Europe: sentinel surveillance data from the European network on surveillance of imported infectious diseases. Clin Infect Dis. 2002;34:572–6. 11803507 10.1086/338235

[45] Danis K Baka A Lenglet A. Autochthonous *Plasmodium vivax* malaria in Greece, 2011. Euro Surveill. 2011;16:19993. 22027375

[46] Checkley AM Smith A Smith V. Risk factors for mortality from imported falciparum malaria in the United Kingdom over 20 years: An observational study. BMJ. 2012;344:e2116. 22454091 10.1136/bmj.e2116 PMC3314185

[47] Cox-Singh J Davis TM Lee KS. *Plasmodium* knowlesi malaria in humans is widely distributed and potentially life threatening. Clin Infect Dis. 2008;46:165–71. 18171245 10.1086/524888 PMC2533694

[48] Mace KE Lucchi NW Tan KR. Malaria surveillance - United States, 2017. MMWR Surveill Summ. 2021;70:1–35. 10.15585/mmwr.ss7002a1 PMC8017932 33735166

[49] Lu G Cao Y Chai L. Barriers to seeking health care among returning travellers with malaria: a systematic review. Trop Med Int Health. 2022;27:28–37. 34748264 10.1111/tmi.13698

[50] Ryan ET Kain KC. Health advice and immunization for travelers. N Engl J Med. 2000;342:1716–25. 10841875 10.1056/NEJM200006083422306

[51] Spira AM. Assessment of travelers who return home ill. Lancet. 2003;361:1459–69. 12727414 10.1016/S0140-6736(03)13141-8

[52] Canadian recommendations for the prevention and treatment of malaria among international travelers. Committee to Advise on Tropical Medicine and Travel CATMAT), Laboratory for Disease Control, 2000. https://pubmed.ncbi.nlm.nih.gov/11055082/. Accessed March 1, 2022. 11055082

[53] Kofoed K Petersen E. The efficacy of chemoprophylaxis against malaria with chloroquine plus proguanil, mefloquine, and atovaquone plus proguanil in travelers from Denmark. J Travel Med. 2003;10:150–4. 12757688 10.2310/7060.2003.35746

[54] Zhou S Li Z Cotter C. Trends of imported malaria in China 2010–2014: analysis of surveillance data. Malar J. 2016;15:39. 26809828 10.1186/s12936-016-1093-0 PMC4727325

[55] Li ZJ Zhang Q Zheng C. Epidemiologic features of overseas imported malaria in the People’s Republic of China. Malar J. 2016;15:141. 26946150 10.1186/s12936-016-1188-7 PMC4779568

[56] Feng J Xiao H Xia Z Zhang L Xiao N. Analysis of malaria epidemiological characteristics in the People’s Republic of China, 2004-2013. Am J Trop Med Hyg. 2015;93:293–9. 26078326 10.4269/ajtmh.14-0733 PMC4530750

[57] Tatem AJ Jia P Ordanovich D. The geography of imported malaria to non-endemic countries: a meta-analysis of nationally reported statistics. Lancet Infect Dis. 2017;17:98–107. 27777030 10.1016/S1473-3099(16)30326-7 PMC5392593

[58] Noor AM Kinyoki DK Mundia CW. The changing risk of *Plasmodium falciparum* malaria infection in Africa: 2000–2010: a spatial and temporal analysis of transmission intensity. Lancet. 2014;383:1739–47. 24559537 10.1016/S0140-6736(13)62566-0 PMC4030588

[59] Yu T Fu Y Kong X Liu X Yan G Wang Y. Epidemiological characteristics of imported malaria in Shandong Province, China, from 2012 to 2017. Sci Rep. 2020;10:7568. 32371895 10.1038/s41598-020-64593-1 PMC7200687

[60] Zhou X Yap P Tanner M Bergquist R Utzinger J Zhou X-N. Surveillance and response systems for elimination of tropical diseases: summary of a thematic series in infectious diseases of poverty. Infect Dis Poverty. 2016;5:49. 27179509 10.1186/s40249-016-0144-7 PMC4868018

[61] Lu G Liu Y Wang J. Malaria training for community health workers in the setting of elimination: a qualitative study from China. Malar J. 2018;17:95. 29475439 10.1186/s12936-018-2229-1 PMC5824442

[62] Shih HH Lin MJ. Long-term impacts of early-life exposure to malaria: Evidence from Taiwan’s Eradication Campaign in the 1950s. Health Econ. 2018;27:1484–512. 29896762 10.1002/hec.3781

[63] World Health Organization. More malaria cases and deaths in 2020 linked to COVID-19 disruptions. 2024. https://www.who.int/news/item/06-12-2021-more-malaria-cases-and-deaths-in-2020-linked-to-covid-19-disruptions. Accessed September 1, 2024.

